# The Mediating Role of Illness Perception Between Psychological Capital and Subjective Well-Being in Breast Cancer Patients: A Cross-Sectional Study

**DOI:** 10.3390/curroncol33100581

**Published:** 2026-09-28

**Authors:** Ming Liu, Ying Zhang, Chengbin Xu, Yu Liu, Yin Wang, Yaxi Wang, Zhuzhu Wang, Jingfang Hong

**Affiliations:** 1 School of Nursing, Anhui Medical University, No. 81 Mei Shan Road, ShuShan District, Hefei 230032, China; 2445011209@stu.ahmu.edu.cn (M.L.);; 2 The First Affiliated Hospital of Anhui Medical University, No. 218 Ji Xi Road, ShuShan District, Hefei 230022, China; 3 Nursing International Collaboration Research Center of Anhui Province, Shushan District, Hefei 230601, China

**Keywords:** breast cancer, psychological capital, illness perception, subjective well-being, mediation analysis

## Abstract

Subjective well-being is increasingly recognized as a key health outcome among breast cancer patients, yet the psychological mechanisms that enhance it remain insufficiently understood. This study aimed to describe the levels of psychological capital, illness perception, and subjective well-being in breast cancer patients, examine their correlations, and test the mediating role of illness perception in the association between psychological capital and subjective well-being. A cross-sectional study was conducted among 256 breast cancer patients recruited by convenience sampling from a provincial tertiary hospital in Hefei, China. Patients were assessed using a self-designed general information questionnaire, the Positive Psychological Capital Questionnaire, the Brief Illness Perception Questionnaire, and the General Well-being Schedule. Data were analyzed using SPSS and PROCESS macro. The mean scores of psychological capital, illness perception, and subjective well-being were 102.31 ± 26.32, 38.54 ± 6.56, and 66.34 ± 14.07, respectively. Pearson’s correlation analysis showed that psychological capital was negatively correlated with illness perception (*r* = −0.648, *p* < 0.01), but positively correlated with subjective well-being (*r* = 0.620, *p* < 0.01). Illness perception was negatively correlated with subjective well-being (*r* = −0.792, *p* < 0.01). Mediation analysis showed that illness perception partially mediated the association between psychological capital and subjective well-being (B = 0.243, 95% CI: 0.194–0.295), accounting for 62.48% of the total effect (B = 0.388, 95% CI: 0.330–0.446). This study underscores the mediating role of illness perception in the association between psychological capital and subjective well-being among breast cancer patients. Reducing negative illness perceptions and enhancing psychological capital may help improve patients’ subjective well-being.

## Simple Summary

Breast cancer patients often experience substantial psychological and physiological challenges during diagnosis and treatment, which can lower their subjective well-being. Subjective well-being reflects a patient’s overall self-evaluation of quality of life and psychological state. Psychological capital is a positive psychological resource composed of self-efficacy, resilience, hope, and optimism. Although evidence suggests that psychological capital is positively associated with subjective well-being, the underlying mechanisms in breast cancer patients remain unclear. Illness perception refers to a patient’s understanding of and emotional response to an illness. This study aimed to examine the mediating role of illness perception in the association between psychological capital and subjective well-being among breast cancer patients. This study surveyed 256 breast cancer patients. Results showed that breast cancer patients reported relatively low levels of subjective well-being, and that illness perception mediated the association between psychological capital and subjective well-being. These findings provide an evidence base for developing targeted interventions to improve subjective well-being in breast cancer patients.

## 1. Introduction

Global Cancer Statistics 2022 reports that breast cancer ranks first in both incidence and mortality among all cancers in women, with approximately 2.309 million new cases and 666,000 deaths [1]. It is projected that by 2070, the number of new breast cancer cases will reach 4.4 million, underscoring the growing challenge that breast cancer poses to women’s health globally [2]. With advances in early diagnosis and multimodal treatment strategies, the prognosis has improved significantly, with corresponding increases in survival rates. Global survival data indicate a 5-year survival rate of 73% and a 10-year survival rate of 61% [3]. As the biopsychosocial model gains wider acceptance in clinical practice, the objectives of treatment have shifted from solely prolonging survival to improving patients’ quality of life and mental health [4].

Breast cancer patients generally require long-term, standardized, and multidisciplinary treatment, throughout which patients frequently experience a range of treatment-related adverse effects, including breast morphological alterations, chemotherapy-induced toxicities, and functional deficits of the affected upper limb [5,6]. Existing studies indicate that approximately 90% of breast cancer patients experience these adverse effects, with 20–50% thereafter presenting anxious and depressive symptoms [7,8]. Moreover, patients have to cope with prognostic uncertainty, challenges in fulfilling family caregiving roles, and the financial burden associated with high treatment costs [9,10,11]. These multifaceted stressors collectively contribute to substantial physical and emotional distress, which may severely compromise patients’ quality of life. Subjective well-being refers to an individual’s overall assessment of their quality of life based on self-defined criteria encompassing both affective and cognitive domains, and serves as a comprehensive indicator of quality of life and psychological state [12]. Research has shown that subjective well-being in cancer patients is associated with enhanced quality of life, reduced psychological distress, and improved symptom experience [13].

Hobfoll’s Conservation of Resources (COR) Theory provides a well-established theoretical framework for explaining the relationship between resources and well-being, suggesting that individuals strive to obtain, keep, and protect the resources they value, and that having sufficient resources contributes to positive emotional experiences and better well-being [14]. Resources refer to elements that individuals value or that help them obtain other valued resources, including personal characteristics, objects, conditions, and energies [14]. A substantial body of research has consistently demonstrated that resources such as core self-evaluation, hope, self-esteem, self-efficacy, optimism, and resilience are positively correlated with subjective well-being [15,16,17]. However, these studies have typically examined only a single type of resource, whereas resources frequently coexist as “resource caravans,” meaning that individuals with one resource tend to simultaneously hold others [18]. As a higher-order positive construct, psychological capital consists of four resources, self-efficacy, resilience, hope, and optimism, and refers to a positive psychological state manifested throughout individuals’ growth and development [19,20]. Researchers have argued that, compared with its individual components, psychological capital as a holistic construct is a stronger predictor of subjective well-being [18,21]. However, a study by Mohsenipouya et al. [22] found that the mechanisms linking psychological capital to subjective well-being remain unclear among breast cancer patients. Thus, although it is widely accepted that psychological capital has a positive effect on subjective well-being [23,24], the mechanisms are still under-explored, and understanding this process is crucial for enhancing patients’ subjective well-being.

Hobfoll [14] posited that an individual’s response to a stressful event depends on their cognitive appraisal, and that this appraisal process is influenced by the resources the individual possesses. Luthans et al. [25] further found that psychological capital can trigger positive cognitive appraisals, drive this process, and serve as a key psychological resource for enhancing subjective well-being. Cognitive appraisal refers to an individual’s evaluation of the nature, severity, and potential harm of a life event they encounter [26]. Leventhal’s Common-Sense Model of Self-Regulation (CSM) proposes that when individuals face a health threat, they form illness perceptions that shape health-related outcomes, including physical, psychological, and functional aspects [27,28]. Illness perception refers to the cognitive and emotional responses that individuals form toward a health threat, building on their prior experiences and the illness-related information they have acquired, and serves as a reflection of patients’ cognitive appraisal [27]. Furthermore, the CSM emphasizes that coping resources can significantly influence an individual’s perceived stress when facing health threats [27,28]. Previous studies have established illness perception as an important mediator through which resources influence psychological adaptation [29,30].

Drawing on the COR theory and CSM, it can be hypothesized that psychological capital, as a coping resource, may facilitate breast cancer patients in developing positive illness perceptions, which in turn may further enhance their subjective well-being. The present study aims to examine the associations among psychological capital, illness perception, and subjective well-being in breast cancer patients, and to test the mediating role of illness perception in the association between psychological capital and subjective well-being, thereby elucidating the underlying psychological mechanism. Based on the aforementioned theoretical models and prior empirical evidence, the following hypotheses are proposed:

**H1.**  *Psychological capital is positively correlated with subjective well-being*.

**H2a.**  *Higher psychological capital is correlated with less negative illness perceptions*.

**H2b.**  *More negative illness perceptions are correlated with lower subjective well-being*.

**H3.**  *Illness perception mediates the association between psychological capital and subjective well-being*.

## 2. Materials and Methods

### 2.1. Participants and Procedure

This study adopted a cross-sectional design and was carried out at a provincial tertiary hospital in Hefei, China. Convenience sampling was used to recruit breast cancer patients admitted to the Departments of Breast Surgery, Medical Oncology, and the Day Chemotherapy Unit between December 2025 and May 2026. The inclusion criteria for participants were as follows: (1) were pathologically diagnosed with breast cancer; (2) were aged 18 years or older; (3) were informed of their diagnosis and condition; (4) were able to communicate or complete the questionnaire. The exclusion criteria were as follows: (1) were diagnosed with a psychiatric or cognitive disorder; (2) were diagnosed with other malignancies or severe life-threatening diseases; (3) were unable to communicate or complete the questionnaire; (4) were currently receiving psychological treatment.

### 2.2. Sample Size

The required sample size was calculated using G*Power (version 3.1), with the following parameters: effect size *f*^2^ = 0.15, alpha error probability = 0.05, power (1–*β* error probability) = 0.90, and number of predictors = 17, yielding a sample size of 179 [31]. Accounting for a 20% rate of invalid responses, at least 224 participants were required, and 256 participants were ultimately included. The post-hoc power analysis for the indirect effect was conducted using Monte Carlo simulation with the bmem package in R (version 4.5.3) [32]. Using the observed path coefficients as population parameters, 1000 datasets were simulated with the current sample size, and the estimated power was 0.839, exceeding the conventional threshold of 0.80 and indicating that the sample size was sufficient.

### 2.3. Data Collection

Trained researchers contacted potential participants only after physicians and nurses had confirmed their clinical stability. All participants were fully informed about the study, and written informed consent was obtained prior to participation. Participants then completed the questionnaire in a quiet and undisturbed environment. A total of 285 participants underwent initial assessment, and 270 of them met the inclusion criteria and completed the questionnaire. The remaining 15 participants were excluded because they either did not meet the inclusion criteria (*n* = 7) or declined to participate (*n* = 8). Privacy concerns and lack of interest were the main reasons for declining to participate. During data screening and eligibility assessment, 14 questionnaires were removed due to more than 15% missing items (*n* = 2) or invalid responses with patterned responding (*n* = 12). Ultimately, 256 participants were included in the final analysis (Figure 1).

### 2.4. Measurements

#### 2.4.1. General Information

The questionnaire was self-developed through deliberations within the research team and encompassed two primary aspects: demographic and clinical characteristics. Demographic characteristics included age (years), partner status, education level, employment status, residence, religious affiliation, monthly per capita household income (CNY), and payment system of medical expenses. Clinical characteristics included disease duration (months), ongoing treatment modality, cancer stage, comorbid conditions, lymph node metastasis, family history of cancer, recruitment department.

#### 2.4.2. Psychological Capital

The Chinese version of the Positive Psychological Capital Questionnaire (PPCQ), validated by Zhang et al. [20], was used to assess patients’ psychological capital and has been applied in previous research [30]. The questionnaire consists of 26 items across four dimensions: self-efficacy (7 items), resilience (7 items), hope (6 items), and optimism (6 items), with each item rated on a seven-point Likert scale (1–7). The total score of the questionnaire ranges from 26 to 182 (items 8, 10, 12, 14, and 25 are reverse-scored), with higher scores indicating more abundant psychological resources. In this study, the Cronbach’s α coefficient of this questionnaire was 0.962.

#### 2.4.3. Illness Perception

The Chinese version of the Brief Illness Perception Questionnaire (BIPQ), validated by Zhang et al. [33] in breast cancer patients, was used to assess patients’ illness perception. The questionnaire consists of 7 items (excluding the open-ended causal item and the illness comprehensibility item) across two dimensions: cognitive (items 1–5), affective (items 6, 8), with each item rated on an eleven-point Likert scale (0–10). The total score of the questionnaire ranges from 0 to 70 (items 3, 4 are reverse-scored), with higher scores indicating more negative illness perceptions. In this study, the Cronbach’s α coefficient of this questionnaire was 0.762.

#### 2.4.4. Subjective Well-Being

The Chinese version of the General Well-being Schedule (GWBS), validated by Duan [34], was used to assess patients’ subjective well-being and has been applied in previous research [35]. The scale consists of 18 items across six dimensions: worry about health (items 10, 15), energy level (items1, 9, 14, and 17), depressed or cheerful mood (items 4, 12, and 18), satisfaction with and interest in life (items 6, 11), relaxation or tension (items 2, 5, 8, and 16), and control over emotional behavior (items 3, 7, and 13). Three different scoring methods were used. Items 2, 5, 6, and 7 were rated on a five-point Likert scale (1–5); items 1, 3, 4, and 8–14 were rated on a six-point Likert scale (1–6); and items 15–18 were rated on an eleven-point Likert scale (0–10). The total score of the schedule ranges from 14 to 120 (items 1, 3, 6, 7, 9, 11, 13, 15, and 16 are reverse-scored), with higher scores indicating better quality of life and higher levels of positive affect. In this study, the Cronbach’s α coefficient of this schedule was 0.948.

### 2.5. Statistical Analysis

Statistical analyses were conducted using SPSS (version 27.0, IBM Corp., Armonk, NY, USA) and the PROCESS macro (version 4.2). All tests were two-tailed, with *p* < 0.05 indicating statistical significance. Normality of distributions was tested using the Kolmogorov–Smirnov test. Continuous variables were presented as mean ± standard deviation (SD), with among-group comparisons performed via Student’s *t*-test or one-way ANOVA. Categorical variables were reported as frequencies and percentages. Pearson’s correlation analysis was performed to examine bivariate correlations among psychological capital, illness perception, and subjective well-being. Variance Inflation Factor (VIF) was used to assess the presence of multicollinearity, with VIF values greater than the conventional threshold of 5 indicating potential multicollinearity.

Mediation analysis was performed via Model 4 of the PROCESS macro to examine the mediation effect of illness perception in the association between psychological capital and subjective well-being. Variables that were significantly associated with subjective well-being in univariate analyses were selected as covariates. The significance of indirect effects was tested using bootstrapping with 5000 resamples, and a 95% confidence interval (CI) that excluded 0 indicated a significant mediation effect. To further assess the stability of the mediation effect, a sensitivity analysis was conducted using Cook’s distance to identify influential observations on model estimates, with a threshold of 4/*N* (*N* = sample size). After excluding these influential cases, the mediation model was re-estimated to examine the consistency of the path coefficients and the mediation effect. The mediation effect proportion was calculated as the ratio of the indirect effect to the total effect.

## 3. Results

### 3.1. Multicollinearity Assessment

The VIF values for all independent variables ranged from 1.132 to 3.372, all below the conventional threshold of 5, indicating that there was no significant multicollinearity issue.

### 3.2. Participant Characteristics and Differences in Subjective Well-Being

The demographic and clinical characteristics of the 256 participants are presented in Table 1. All participants were female, with a mean age of 54.00 years. The majority of participants were partnered (91.8%), had an education level of elementary school or below (43.0%), lived in urban areas (38.7%), and were unemployed (39.1%). A minority of participants reported religious affiliation (5.9%). Regarding monthly household income, 48.0% reported earning between 3000 and 4999 CNY per capita. The most common ongoing treatment modality was surgery (48.4%), and the predominant medical insurance type was urban and rural resident medical insurance (68.8%). In terms of disease stage, 51.2% of participants were classified as stage II, and the majority had no lymph node metastasis (55.1%), had comorbidities (54.7%), and had no family history of cancer (89.9%). As for disease duration, the majority of participants (64.1%) had a duration of less than 3 months. In addition, 39.1% of participants were recruited from the Department of Breast Surgery.

Univariate analyses indicated that subjective well-being scores varied significantly by age, partner status, employment status, religious affiliation, monthly per capita household income, disease duration, and ongoing treatment modality (all *p* < 0.05).

### 3.3. Mean Scores and Correlations for Psychological Capital, Illness Perception, and Subjective Well-Being

As depicted in Table 2, the mean scores of psychological capital, illness perception, and subjective well-being were 102.31 ± 26.32, 38.54 ± 6.56, and 66.34 ± 14.07, respectively. Pearson’s correlation analysis showed that psychological capital was negatively correlated with illness perception (*r* = −0.648, *p* < 0.01), but positively correlated with subjective well-being (*r* = 0.620, *p* < 0.01). Illness perception was negatively correlated with subjective well-being (*r* = −0.792, *p* < 0.01).

### 3.4. Mediating Effects of Illness Perception Between Psychological Capital and Subjective Well-Being

Psychological capital served as the independent variable and subjective well-being as the dependent variable, with age, partner status, employment status, religious affiliation, monthly per capita household income, disease duration, and ongoing treatment modality as covariates. Illness perception was examined as a mediator in the association between psychological capital and subjective well-being.

Coding schemes for all categorical variables were detailed in Table 3. Regression analysis results are presented in Table 4. Psychological capital showed a positive association with subjective well-being (*β* = 0.726, *p* < 0.001). Even after the inclusion of illness perception, psychological capital remained significantly positively associated with subjective well-being (*β* = 0.272, *p* < 0.001). Furthermore, psychological capital was negatively associated with illness perception (*β* = −0.733, *p* < 0.001), while illness perception was negatively associated with subjective well-being (*β* = −0.619, *p* < 0.001).

As presented in Table 5, the mediation analysis showed that illness perception partially mediated the association between psychological capital and subjective well-being (B = 0.243, 95% CI: 0.194–0.295), accounting for 62.48% of the total effect (B = 0.388, 95% CI: 0.330–0.446). The specific pathways depicting the interrelationships among these variables are presented in Figure 2. To further assess the stability of the mediation effect, a sensitivity analysis was conducted. After removing 17 influential cases identified by Cook’s distance, 239 participants remained for re-estimation (Supplementary Table S1). In the re-estimated model, the magnitude of all path coefficients changed only marginally. The indirect effect remained significant (B = 0.254, 95% CI: 0.206–0.307), accounting for 63.94% of the total effect (B = 0.397, 95% CI: 0.340–0.454), indicating that the mediation effect was robust.

## 4. Discussion

In this study, the score for subjective well-being among breast cancer patients was slightly lower than the score reported by Yu et al. [35] among breast cancer patients undergoing chemotherapy. This result may be attributed to the relatively high proportion of patients who underwent surgery in our sample (48.4%), physical symptoms resulting from surgery—such as pain, fatigue, and functional impairment—may create a persistent psychological and physiological burden that weakens positive emotional experiences and affects patients’ subjective well-being [6]. Additionally, as regardless of whether breast-conserving surgery or mastectomy was performed, breast appearance changes can impair patients’ body image and satisfaction with their physical appearance [36]. In cultural contexts where femininity, sexuality and family roles are closely linked to women’s bodily integrity and social identity, changes in breast appearance may be associated with reduced self-esteem, altered identity and increased stigma [37]. Another possible explanation is that most participants were in the early stage of diagnosis and treatment. According to hedonic adaptation theory, major life events may temporarily disrupt well-being before individuals gradually adapt over time [38]. In this study, 64.1% of participants had been diagnosed for less than three months, suggesting that many had not yet had sufficient time to adjust emotionally and cognitively to the disease. This temporal pattern is consistent with Suh et al. [39], who concluded that the impact of most life events, particularly negative ones, on subjective well-being diminishes in less than three months.

Breast cancer patients exhibited a relatively negative perception of their illness compared with lung cancer patients [40], a discrepancy that may be related to differences in cancer types and treatment context. This may also involve the role of sex hormones in emotional regulation, with evidence suggesting that fluctuations in oestradiol and progesterone can increase emotional vulnerability [41]. The psychological capital level of breast cancer patients was moderate-to-low, consistent with the findings of Guo et al. [30], which may be attributed to the multiple psychosocial stressors encountered during treatment-including changes in self-image, shifts in family roles, and increased financial burdens-that collectively deplete patients’ psychological resources [42].

This study identified illness perception as a significant partial mediator in the association between psychological capital and subjective well-being, accounting for 62.48% of the total effect. The substantial proportion indicates that the association between psychological capital and subjective well-being was largely explained by patients’ perceptions of their illness, rather than by a direct pathway alone. As a positive psychological resource, psychological capital may contribute to more adaptive appraisals of illness, including the view that the illness is manageable and treatable [25,30]. Illness perception is closely associated with patients’ subjective well-being, as positive illness perceptions reduce their fear and helplessness about the future, increase positive emotional experiences, improve their evaluations of life satisfaction, and thus enhance their subjective well-being [29,43,44]. The present study confirms the applicability of an integrated theoretical framework combining COR theory and CSM in elucidating the underlying mechanisms for how illness perception mediates the association between psychological capital and subjective well-being in breast cancer patients. Furthermore, the association between psychological capital and subjective well-being remained after illness perception was included. From the perspective of COR theory, this direct effect can be explained by the fact that the more abundant a patient’s psychological resources are, the stronger their inner belief in overcoming the illness, the more confident they are in defeating the disease, the more optimistic they remain about the treatment process, and the more hope they have for their future life, all of which collectively help patients maintain a positive psychological state [14].

This finding is consistent with previous research demonstrating the mediating role of illness perception in linking psychological resources to well-being. Among parents of children with cancer, resources such as hope and social support have been found to indirectly enhance their well-being by improving their illness perceptions [29]. Notably, this mediating role demonstrated relevance beyond oncology, supporting the concept of its trans-diagnostic utility. Among military personnel exposed to trauma, trait psychological capital (resilience, hope, and optimism) partially mediates its impact on health symptoms (anxiety, depression, and somatization) through cognitive appraisals [45]. These consistent findings across diverse populations suggest that cognitive appraisals may serve as an important and modifiable psychological pathway in mediating the association between resources and health outcomes.

### 4.1. Clinical Implications

These findings highlight the potential value of incorporating assessments of psychological capital and illness perception into routine clinical evaluations for breast cancer patients. Particular attention should be given to patients with low psychological capital and negative illness perception, as they may benefit most from targeted support. For patients with negative illness perceptions, healthcare professionals can help them develop more coherent, controllable, and less threatening understandings of breast cancer. The CSM provides a framework for achieving this [46]. This can begin with providing systematic information about breast cancer, including its causes, symptoms, treatment modalities, side effects, and prognosis, which helps patients build a coherent understanding of their illness. It can be followed by explaining the purpose and expected effects of treatment, together with postoperative functional exercise plans or chemotherapy self-management checklists, which can strengthen patients’ treatment and personal control perceptions. It can be further supported by organizing activities such as mindfulness meditation, music therapy, and written emotional disclosure to promote reciprocal communication among patients, which can help them express and regulate illness-related concerns and emotional responses, thereby supporting more adaptive adjustment throughout the treatment trajectory. In addition, psychological capital interventions may still be valuable, as they can help patients develop more adaptive illness perceptions. The Psychological Capital Intervention (PCI), developed by Luthans et al. [47], is an intervention model that targets four aspects: self-efficacy, resilience, hope, and optimism. Healthcare professionals can use this framework to guide patients in setting valuable and challenging goals and planning multiple pathways to achieve them, with modeling and social persuasion used to strengthen patients’ confidence and positive expectations.

### 4.2. Limitations and Future Direction

Several limitations of this study should be acknowledged when interpreting the results. First, the cross-sectional design precludes causal inference, as all variables were assessed simultaneously, making it impossible to determine the temporal order of the observed relationships. Second, the use of convenience sampling from a single tertiary hospital may limit the generalizability of the findings and introduce potential selection bias. Third, although the current sample size was adequate for the primary analyses, it may still be insufficient for testing more complex models. Fourth, although we adjusted for a range of covariates, the possibility of residual confounding due to unmeasured factors cannot be completely excluded. Finally, the reliance on self-reported measures is subject to potential biases. Several directions for future research can be proposed in light of these limitations. First, future studies could use a longitudinal design to measure psychological capital, illness perception, and subjective well-being at multiple time points in order to determine the temporal order of changes among these three variables and to examine whether the mediating role of illness perception remains stable across different stages. Second, future studies could adopt multi-center designs with larger samples and include additional relevant factors, in order to more rigorously test the mediating pathways, obtain more stable parameter estimates, and reduce residual confounding.

## 5. Conclusions

This study found significant associations among psychological capital, illness perception, and subjective well-being in breast cancer patients. Illness perception mediated the association between psychological capital and subjective well-being. These findings provide empirical evidence for understanding the mediating role of cognitive pathways in the association between psychological resources and mental health outcomes in breast cancer patients, and offer guidance for developing targeted psychological interventions. Both psychological capital and maladaptive illness perceptions represent potential targets for supportive interventions. Enhancing psychological capital and promoting a more objective and manageable appraisal of illness may ultimately improve patient well-being.

## Figures and Tables

**Figure 1:**
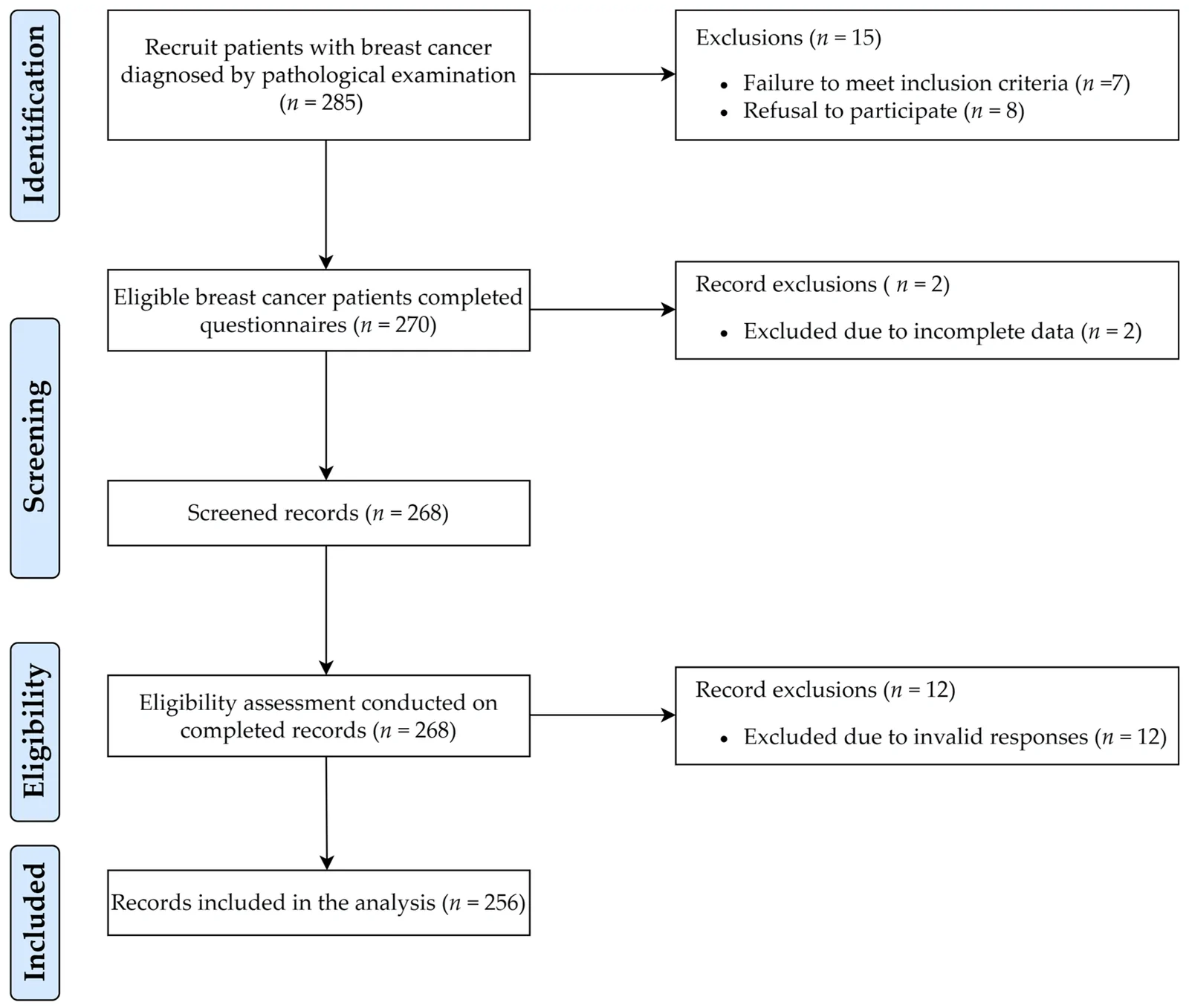
Study recruitment flowchart.

**Figure 2:**
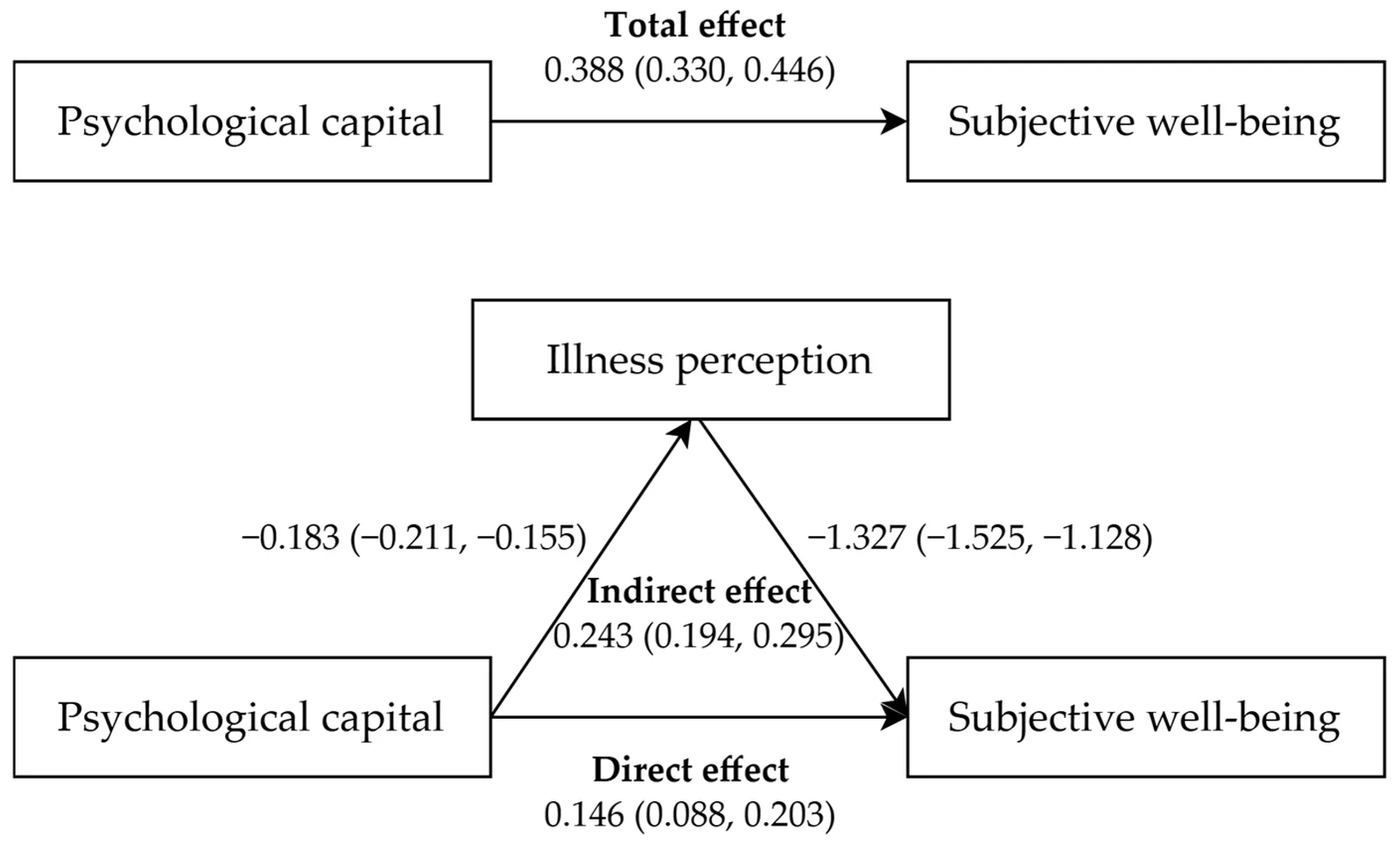
The mediating role of illness perception between psychological capital and subjective well-being in breast cancer patients.Unstandardized path coefficients (B) and their 95% confidence intervals (CIs) are presented.

**Table 1:** General characteristics of participants and distribution of subjective well-being scores (*N* = 256).

Variable	*N* (%)	Mean ± SD	t/F	*p* Values
Age (years)			4.794 ^b^	0.009
<40	30 (11.7)	59.90 ± 11.96		
40–59	151 (59.0)	66.23 ± 14.23		
≥60	75 (29.3)	69.16 ± 13.81		
Partner status			2.256 ^a^	0.025
With partner	235 (91.8)	66.93 ± 13.83		
Without partner	21 (8.2)	59.76 ± 15.32		
Education level			1.149 ^b^	0.319
Elementary school and below	110 (43.0)	65.49 ± 14.05		
Junior high school	84 (32.8)	65.73 ± 13.05		
High school and above	62 (24.2)	68.69 ± 15.34		
Employment status			2.722 ^b^	0.045
Unemployed	100 (39.1)	64.11 ± 14.11		
Farmer	42 (16.4)	65.33 ± 12.77		
Employed	73 (28.5)	67.23 ± 14.50		
Retired	41 (16.0)	71.24 ± 13.57		
Residence			2.356 ^b^	0.097
Urban	99 (38.7)	68.73 ± 15.01		
Town	86 (33.6)	65.01 ± 12.99		
Rural	71 (27.7)	64.63 ± 13.69		
Religious affiliation			2.247 ^a^	0.026
Yes	15 (5.9)	74.20 ± 12.44		
No	241 (94.1)	65.85 ± 14.04		
Monthly per capita household income (CNY)			8.953 ^b^	<0.001
<3000	50 (19.5)	63.72 ± 13.44		
3000–4999	123 (48.0)	63.89 ± 12.68		
>5000	83 (32.4)	71.55 ± 15.09		
Payment system of medical expenses			1.320 ^a^	0.188
Urban and rural resident medical insurance	176 (68.8)	65.56 ± 14.31		
Employee medical insurance	80 (31.3)	68.06 ± 13.45		
Disease duration (months)			3.445 ^a^	<0.001
<3	164 (64.1)	64.12 ± 13.76		
≥3	92 (35.9)	70.30 ± 13.81		
Ongoing treatment modality			9.956 ^b^	<0.001
Surgery	124 (48.4)	63.77 ± 13.94		
Chemotherapy	84 (32.8)	67.10 ± 13.29		
Targeted therapy	16 (6.3)	83.06 ± 10.18		
Chemotherapy + Targeted therapy	32 (12.5)	66.00 ± 12.70		
Cancer stage			0.596 ^b^	0.618
0	17 (6.6)	66.35 ± 14.23		
I	73 (28.5)	66.89 ± 13.50		
II	131 (51.2)	66.82 ± 14.33		
III	35 (13.7)	63.40 ± 14.41		
Comorbid conditions			0.473 ^a^	0.636
Yes	140 (54.7)	65.96 ± 14.64		
No	116 (45.3)	66.80 ± 13.40		
Lymph node metastasis			0.067 ^a^	0.947
Yes	115 (44.9)	66.41 ± 13.78		
No	141 (55.1)	66.29 ± 14.35		
Family history of cancer			0.999 ^a^	0.319
Yes	26 (10.2)	63.73 ± 11.43		
No	230 (89.9)	66.64 ± 14.33		
Recruitment department			2.567 ^b^	0.079
Breast Surgery	100 (39.1)	65.18 ± 13.79		
Medical Oncology	57 (22.3)	64.14 ± 14.27		
Day Chemotherapy Unit	99 (38.7)	68.79 ± 14.01		

Note: ^a^ = Student’s *t*-test, ^b^ = one-way ANOVA.

**Table 2:** Mean scores and correlations among variables.

Variable	Mean ± SD	1	2	3
1. Psychological capital	102.31 ± 26.32	1		
2. Illness perception	38.54 ± 6.56	−0.648 **	1	
3. Subjective well-being	66.34 ± 14.07	0.620 **	−0.792 **	1

Note: ** *p* < 0.01.

**Table 3:** General information variable assignment table.

Variable	Encoding (Dummy Variable Setup)
Age (years)	Dummy1: 1 = 40–59; 0 = ≥60; 0 = <40 (Reference)
	Dummy2: 0 = 40–59; 1 = ≥60; 0 = <40 (Reference)
Partner status	1 = With partner; 0 = Without partner (Reference)
Employment status	Dummy1: 1 = Farmer; 0 = Employed; 0 = Retired; 0 = Unemployed (Reference)
	Dummy2: 0 = Farmer; 1 = Employed; 0 = Retired; 0 = Unemployed (Reference)
	Dummy3: 0 = Farmer; 0 = Employed; 1 = Retired; 0 = Unemployed (Reference)
Religious affiliation	1 = Yes; 0 = No (Reference)
Monthly per capita household income (CNY)	Dummy1: 1 = 3000–4999; 0 = ≥5000; 0 = <3000 (Reference)
	Dummy2: 0 = 3000–4999; 1 = ≥5000; 0 = <3000 (Reference)
Disease duration (months)	1 = ≥3; 0 = <3 (Reference)
Treatment modality	Dummy1: 1 = Chemotherapy; 0 = Targeted therapy; 0 = Chemotherapy + Targeted therapy; 0 = Surgery (Reference)
	Dummy2: 0 = Chemotherapy; 1 = Targeted therapy; 0 = Chemotherapy + Targeted therapy; 0 = Surgery (Reference)
	Dummy3: 0 = Chemotherapy; 0 = Targeted therapy; 1 = Chemotherapy + Targeted therapy; 0 = Surgery (Reference)

**Table 4:** Regression analysis for mediation model.

Variable	Subjective Well-Being				Illness Perception				Subjective Well-Being			
	B (95% CI)	SE	*β*	*p*	B (95% CI)	SE	*β*	*p*	B (95% CI)	SE	*β*	*p*
(Constant)	21.386 (14.015, 28.757)	3.742		<0.001	60.134 (56.538, 63.731)	1.826		<0.001	101.174 (87.970, 114.377)	6.703		<0.001
Age (ref: <40)												
40–59	4.255 (0.304, 8.206)	2.006	0.149	0.035	−2.626 (−4.554, −0.698)	0.979	−0.197	0.008	0.772 (−2.291, 3.834)	1.555	0.027	0.620
≥60	7.759 (3.227, 12.290)	2.300	0.252	<0.001	−3.880 (−6.091, −1.668)	1.123	−0.270	<0.001	2.611 (−0.934, 6.157)	1.800	0.085	0.148
Partner status (ref: Without partner)	4.927 (0.408, 9.447)	2.295	0.096	0.033	−3.306 (−5.511, −1.100)	1.120	−0.139	0.004	0.542 (−2.972, 4.055)	1.784	0.011	0.762
Employment status (ref: Unemployed)												
Farmer	−1.255 (−4.995, 2.484)	1.898	−0.033	0.509	0.984 (−0.841, 2.808)	0.926	0.056	0.289	0.050 (−2.812, 2.913)	1.453	0.001	0.973
Employed	−3.196 (−6.678, 0.287)	1.768	−0.103	0.072	0.972 (−0.727, 2.672)	0.863	0.067	0.261	−1.906 (−4.573, 0.761)	1.354	−0.061	0.161
Retired	−4.127 (−8.281, 0.027)	2.109	−0.108	0.052	1.760 (−0.267, 3.787)	1.029	0.099	0.089	−1.792 (−4.984, 1.400)	1.620	−0.047	0.270
Religious affiliation (ref: No)	5.244 (−0.076, 10.565)	2.701	0.088	0.053	−2.054 (−4.650, 0.542)	1.318	−0.074	0.121	2.519 (−1.564, 6.603)	2.073	0.042	0.225
Monthly per capita household income (ref: <3000)												
3000–4999	−6.698 (−10.270, −3.126)	1.813	−0.238	<0.001	2.087 (0.344, 3.830)	0.885	0.159	0.019	−3.928 (−6.688, −1.169)	1.401	−0.140	0.006
≥5000	−7.180 (−11.746, −2.614)	2.318	−0.239	0.002	2.963 (0.735, 5.191)	1.131	0.212	0.009	−3.249 (−6.785, 0.288)	1.795	−0.108	0.072
Disease duration (ref: <3)	0.839 (−2.842, 4.520)	1.869	0.029	0.654	1.680 (−0.117, 3.476)	0.912	0.123	0.067	3.067 (0.236, 5.899)	1.437	0.105	0.034
Treatment modality (ref: Surgery)												
Chemotherapy	4.626 (1.049, 8.202)	1.816	0.155	0.012	−0.640 (−2.385, 1.106)	0.886	−0.046	0.471	3.777 (1.043, 6.512)	1.388	0.126	0.007
Targeted therapy	15.817 (9.334, 22.299)	3.291	0.273	<0.001	−4.003 (−7.166, −0.839)	1.606	−0.148	0.013	10.506 (5.491, 15.520)	2.546	0.181	<0.001
Chemotherapy + Targeted therapy	1.229 (−3.673, 6.132)	2.489	0.029	0.622	0.961 (−1.431, 3.353)	1.214	0.049	0.430	2.504 (−1.245, 6.254)	1.903	0.059	0.190
Psychological capital	0.388 (0.330, 0.446)	0.029	0.726	<0.001	−0.183 (−0.211, −0.155)	0.014	−0.733	<0.001	0.146 (0.088, 0.203)	0.029	0.272	<0.001
Illness perception									−1.327 (−1.525, −1.128)	0.101	−0.619	<0.001
R^2^	0.561				0.519				0.745			
Adj. R^2^	0.536				0.491				0.729			
F	22.002				18.589				46.752			
*p*	<0.001				<0.001				<0.001			

**Table 5:** Path coefficients and mediation effects of illness perception on the relationship between psychological capital and subjective well-being in breast cancer patients (*N* = 256).

Effect	B	SE	95% CI		*p*	Relative Effect %
			LLCI	ULCI		
Total effect						
c Psychological capital → Subjective well-being	0.388	0.029	0.330	0.446	<0.001	
Direct effect						37.52
c′ Psychological capital → Subjective well-being	0.146	0.029	0.088	0.203	<0.001	
Indirect effect						62.48
a Psychological capital → Illness perception	−0.183	0.014	−0.211	−0.155	<0.001	
b Illness perception → Subjective well-being	−1.327	0.101	−1.525	−1.128	<0.001	
a × b	0.243	0.026	0.194	0.295	<0.001	

## Data Availability

The data presented in this study are available on request from the corresponding author.

## Supplementary Materials

The following supporting information can be downloaded at https://www.mdpi.com/article/10.3390/curroncol33100581/s1, Table S1. Sensitivity analysis of the mediation model after excluding influential observations identified via Cook’s distance (*N* = 239).
