# Supplementary material for: The Mediating Role of Illness Perception Between Psychological Capital and Subjective Well-Being in Breast Cancer Patients: A Cross-Sectional Study
Source: Curr Oncol. 2026 Sep 28;33(10):581. doi: 10.3390/curroncol33100581 (PMC13703243; doi:10.3390/curroncol33100581)
Supplement: Supplementary file 1 [file curroncol-33-00581-s001.zip › curroncol-4528300-supplementary.pdf]

Supplementary Table S1. Sensitivity analysis of the mediation model after excluding influential observations identified via Cook's distance (*N* = 239).

Effect	B	SE	95% *CI*		*P*	Relative Effect %
			LLCI	ULCI		
Total effect						
c Psychological capital → Subjective well-being	0.397	0.029	0.340	0.454	< 0.001	
Direct effect						36.03
c' Psychological capital→ Subjective well-being	0.143	0.025	0.094	0.192	< 0.001	
Indirect effect						63.94
a Psychological capital → Illness perception	-0.180	0.015	-0.210	-0.150	< 0.001	
b Illness perception → Subjective well-being	-1.410	0.086	-1.579	-1.241	< 0.001	
a*b	0.254	0.026	0.206	0.307	< 0.001	
